# Investigating risk profiles of smartphone activities and psychosocial factors in adolescents during the COVID‐19 pandemic

**DOI:** 10.1111/jora.13045

**Published:** 2024-12-29

**Authors:** Ann‐Christin Haag, Elizabeth A. Nick, Mark S. Chen, Eva H. Telzer, Mitchell J. Prinstein, George A. Bonanno

**Affiliations:** ^1^ Teachers College, Columbia University, Department of Counseling and Clinical Psychology, Teachers College Columbia University New York New York USA; ^2^ Department of Child and Adolescent Psychiatry/Psychotherapy University of Ulm Ulm Germany; ^3^ Partner Site Mannheim‐Heidelberg‐Ulm German Center for Mental Health (DZPG) Ulm Germany; ^4^ Department of Psychology and Neuroscience University of North Carolina at Chapel Hill Chapel Hill North Carolina USA; ^5^ Department of Psychology Yale University New Haven Connecticut USA

**Keywords:** adolescents, risk profiles, smartphone use, social media, well‐being & mental health

## Abstract

Associations of adolescents' smartphone use and well‐being have been contradictory. The present study investigates patterns of smartphone use and psychosocial risk / protective factors in US adolescents during COVID‐19 and examines their associations with depression symptom trajectories from 5 yearly waves beginning prior to the COVID‐19 pandemic. Latent profile analyses revealed three risk profiles, including a high risk profile (18.9% adolescents) characterized by elevated social media use, high levels of psychosocial risk, and low levels of protective variables. Latent growth mixture modeling identified three depression trajectories; stable low, moderate‐increasing, and high‐severely increasing depression. Both the moderate‐increasing and high‐severely increasing depression trajectories were associated with membership in the high risk profile. Results highlight the impacts of type of smartphone activity rather than use per se and can inform targeted intervention strategies.

## INTRODUCTION

Smartphones represent an integral part of young people's lives today, with the vast majority of adolescents owning or having access to a smartphone. In studies of nationally representative samples, 45% of adolescents reported that they are online “almost constantly” and 44% several times per day (Anderson & Jiang, 2018). However, associations of smartphone use with well‐being have been inconsistent and suffered from methodical and conceptual limitations. The present study attempted to advance existing research by investigating patterns of objectively assessed smartphone use and psychosocial risk versus protective factors using a person‐centered approach in a sample of US adolescents during COVID‐19 and by examining their association with trajectories of depressive symptoms from 5 yearly waves of data beginning prior to and continuing during the COVID‐19 pandemic.

### Smartphone use and adolescent well‐being

The ubiquity of smartphones amongst adolescents has led to considerable debate about their potential implications for development and well‐being. However, the number of studies specifically investigating smartphone use is still comparatively restricted. Instead, researchers have often studied the broader umbrella concept of screen time, that is, time spent using any kind of screen device, including for example, television, computers, or smartphones. Findings on adolescents' screen time have been contradictory, with some studies associating elevated screen time with a variety of health costs, including adiposity, depressive symptoms, and quality of life (reviewed in Stiglic & Viner, 2019), while others revealed only small or null effects regarding mental health (reviewed in Odgers & Jensen, 2020; Orben, 2020) and behavior problems (reviewed in Eirich et al., 2022). However, researchers raised serious methodological concerns about the measurement of screen time, including poor conceptualization, relying on self‐report measures, and difficulties when measuring screen time over time and across varying contexts (Kaye et al., 2020). Specifically examining smartphone use, a meta‐analysis including over 41 studies on children and young people (<25 years) revealed an association of smartphone use mapping onto concepts of addiction with increased odds of depression, increased anxiety, higher perceived stress, and poorer sleep quality (Sohn et al., 2019). In addition, lowered quality of peer relationship was associated with addictive smartphone use (Su et al., 2022). More recently, a study identifying clusters of adolescents, has provided evidence for greater objectively traced smartphone use being linked to greater levels of distress (Fortunato et al., 2023).

Relatedly, theoretical frameworks have emphasized various psychosocial risk and protective factors. They highlight the importance of more deeply understanding adolescents' smartphone use by taking into account a comprehensive set of psychosocial variables related to adolescent development and well‐being. The *Interaction of Person‐Affect‐Cognition‐Execution* (*I‐PACE*) *model* (Brand et al., 2019), for example, postulates that certain patterns of addictive use of internet applications, and thereby presumably also smartphone applications, are associated with specific personal characteristics coupled with individual gratification processes, which can be related to specific vulnerabilities and various psychopathological outcomes. In addition, the *Differential Susceptibility to Media Effects Model* (DSMM; Valkenburg & Peter, 2013) suggests that dispositional (e.g., motivations, moods) and social (e.g., parents, peers) dimensions as well as developmental stages may either predict selective use of digital technology, including smartphone use, or moderate its effect on cognitive and emotional outcomes. Finally, focusing specifically on social media as a new interpersonal context for youth, the *transformational framework* (Nesi et al., 2018) highlights the importance of peer relationships, but also their transformation by social media.

### Social media use and adolescent well‐being

Narrowing down the focus to one application of smartphones, studies have extensively researched impacts of social media use on adolescents, since adolescents spend much of their time on social media when using their smartphones (almost one‐third of smartphone time is spent using social media as per the results of the present study). Studies of adolescents' subjective reports of smartphone use have revealed that teens perceive their smartphone use as frequent, especially to facilitate social media use (Anderson & Jiang, 2018) and report about 1.27 h of social media use per day (Rideout et al., 2022). While there certainly are possible benefits for adolescents in using social media, like social connection (e.g., Clark et al., 2018), creative expression, and shared humor (e.g., Eales et al., 2023), there is also considerable evidence documenting its detrimental impact on mental health and well‐being (e.g., reviewed in Schønning et al., 2020). As such, elevated social media use has been linked to increased levels of depression, anxiety as well as suicidality (Gingras et al., 2023; Nesi, Rothenberg, et al., 2021; Piteo & Ward, 2020; Vannucci & McCauley Ohannessian, 2019; Vidal et al., 2020), decreased amount and quality of sleep (e.g., Nesi, Burke, et al., 2021), lower self‐esteem (Woods & Scott, 2016), and lower levels of happiness (Twigg et al., 2020). Finally, elevated psychosocial risk factors, such as risk‐taking behaviors (Nesi & Prinstein, 2015; Vannucci & McCauley Ohannessian, 2019), as well as lowered protective factors, including diminished family and friend support (Noll et al., 2021; Vannucci & McCauley Ohannessian, 2019) have been revealed to be associated with increased social media use.

Studies on impacts of social media use have heavily focused on depression as an outcome of mental well‐being (Schønning et al., 2020). Multiple reasons can explain this focus. First, parallel to the increasing popularity of social media use (Anderson & Jiang, 2018), prevalence rates of depression have also risen over the past decade (Twenge & Campbell, 2019). Second, with a lifetime prevalence of 11.7%, major depressive disorder is one of the most common and costly mental disorders amongst adolescents (Merikangas et al., 2010). Nonetheless, findings remain inconsistent. While some researchers report a positive association between social media use (Keles et al., 2020; McCrae et al., 2017; Twenge & Campbell, 2019) and depressive symptoms, other studies reveal null associations (Coyne et al., 2020; Kreski et al., 2021; Steinsbekk et al., 2023) or associations too small to be of clinical relevance (Orben, 2020; Orben & Przybylski, 2019). In addition, in a recent longitudinal study, Gingras et al. (2023) did not find depression to be predictive of social media use 1 year later. Hence, there is a need to further disentangle the directionality of the relationship of depression and social media use, and, more broadly, smartphone use.

### Effects of the COVID‐19 pandemic

The COVID‐19 pandemic presented an unusually challenging stressor context for both adolescent well‐being and smartphone use. Recently published reviews and meta‐analyses including studies throughout the world describe a rise of mental health symptoms in youth during COVID‐19, such as distress, depression, anxiety, eating disorders, substance use, self‐harm, and suicidality (Goldstein, 2023; Jones et al., 2021; Viner et al., 2022; Wang et al., 2022). For example, a study analyzing over 45,000 emergency visits found that the frequency of anxiety and depression significantly increased from the pre‐COVID‐19 to the COVID‐19 period in a sample of US adolescents and young adults (Workman et al., 2023). At the same time, reviews highlight increases in screen time and social media use in adolescents during COVID‐19 (Goldstein, 2023; Viner et al., 2022). The mean daily screen time amongst young US adolescents more than doubled during COVID‐19, jumping from 3.8 h daily to 7.70 h daily, encompassing mostly viewing videos, movies, or television shows. Further, higher screen use was associated with poorer mental health and greater perceived stress during COVID‐19 (Nagata et al., 2022). Similarly, increases in smartphone use and increased levels of stress in adolescents have been reported during the COVID‐19 pandemic compared to pre‐COVID‐19 levels (Nutley et al., 2023). Finally, elevated rates of social media use have been shown in adolescents, coupled with experiencing COVID‐19‐related anxiety (Kim et al., 2023).

### Trajectories of adjustment across adolescence

Both research and theory (Bonanno, 2004; Bonanno et al., 2023) have argued that responses to aversive life events go beyond a simple binary distinction of psychopathology versus the absence of psychopathology. Rather, these responses are heterogeneous and can be captured by multiple prototypical outcome trajectories. Studies examining heterogeneity in longitudinal courses of symptoms after stressful life events have adopted sophisticated statistical approaches, including person‐centered approaches, such as latent growth mixture modeling (LGMM), which allows for the identification of latent distributions within the larger sample based on similarities in degree of symptom severity and change over time. The most common prospective and longitudinal trajectories, observed in almost 100 studies of adults after adverse events, including the COVID‐19 pandemic, are chronic symptoms elevations, acute symptoms followed by recovery, delayed increases in symptoms, and the modal response across studies, a stable trajectory of healthy adjustment or resilience (for reviews, see Galatzer‐Levy et al., 2018; Schäfer et al., 2022). This approach has also been applied to identify longitudinal patterns of symptoms across development in children and adolescents (e.g., Haag et al., 2022). A recent meta‐analysis (Shore et al., 2018) reported consistent evidence of heterogeneous trajectories of depressive symptoms in children and adolescents characterized by “No or low,” “Moderate,” “High,” “Increasing,” and “Decreasing” depressive symptoms over time. Consistent with studies of adults, the majority, 56% of the child and adolescent samples included in this analysis (*N* = 41,236) were represented in “No or low” depressive symptom trajectories.

### Methodological limitations of existing research

One limitation of existing research examining overall screen time is that it may mask more heterogeneous patterns. For example, different types of most frequent, objectively assessed online activities, such as social media or entertainment, can hold unique implications for adolescent well‐being (Noll et al., 2021). It would, therefore, be fruitful to apply a person‐centered approach to investigate patterns of most frequent types of smartphone activities in combination with an array of individual and social risk and protective factors. This approach would facilitate the identification of both health and at‐risk profiles of adolescents while also taking into account how developmental context contributes to smartphone use patterns (Eales et al., 2023). Finally, much of the existing research on smartphone use has relied on self‐report and has been conducted at a single time point, thus limiting causal conclusions on whether technology use precedes and/or predicts mental health outcomes or vice versa. As such, more longitudinal research is needed to elucidate the relationship between patterns of frequent types of smartphone use and adolescent well‐being in order to gain a better understanding about the directionality of effects.

### The current study

In this current study, seeking to incorporate smartphone use within a more holistic developmental framework, we aimed to address these limitations and further disentangle the relationship between types of smartphone activities and psychosocial risk versus protective factors in two ways. First, we objectively assessed and described different patterns of most frequent smartphone activities in youth by investigating their links to a diverse set of both psychosocial risk and protective factors. Accordingly, we adopted a person‐centered approach, latent profile analysis (LPA), to empirically derive distinct profiles based on the confluence of adolescent's most frequent smartphone activities coupled with coexisting psychosocial risk and protective factors. This approach allowed us to probe whether distinct subgroups might exhibit different types of frequent smartphone use coupled with different sets of either risk or protective factors. Given the implications reviewed above, we were particularly interested in investigating social media use versus other types of most frequent uses (e.g., video streaming). Based on the existing literature detailed above, we chose a broad array of both psychosocial risk and protective factors that have been both theoretically and empirically associated with smartphone use, or screen time in more general. Expanding on established risk or protective factors, we further included factors that represent topics of particular relevance during adolescence from a developmental perspective, that is, stress, risk taking, and relationship with caregivers and peers (e.g., Blakemore, 2019; Blankenstein et al., 2020). In addition, due to the special circumstances under which this study took place, we also included COVID‐19‐related emotional outcomes as risk factors. By investigating all potential risk and protective factors described above jointly in the person‐centered analytic approach of the present study, we were leveraging the advantages of unsupervised machine‐learning techniques like LPA, using a comprehensive set of relevant variables to create a fine‐grained picture of unique patterns and associations characterizing subgroups of adolescents.

The second key feature of the current study was that we examined the relationship of the latent profiles of smartphone use and risk and protective factors to longer term trajectories of adjustment. We identified trajectories of depression beginning 4 years prior to and culminating during the first year of the COVID‐19 pandemic. We then examined predictive associations of these trajectories with the latent profiles of smartphone use and psychosocial risk and/or protective factors during the pandemic.

Based on the theoretical and empirical literature cited above demonstrating how social media use can disrupt normal adolescent development (e.g., Nesi et al., 2018; Schønning et al., 2020), risk profiles which include elevated rates of social media use were of particular interest. As such, our analyses were guided by two sets of hypotheses.

1. The profiles identified by LPA will include a comparably small, but critical subgroup of high‐risk adolescents characterized by elevated social media use coupled with multiple psychosocial risk factors, specifically greater COVID‐19‐related negative emotions and worries, perceived stress, risk‐taking behaviors, more frequent negative interactions with parents and peers, and few protective factors, specifically lowered support by parents and peers. LPA will further reveal larger subgroups of adolescents characterized by other most frequent smartphone activities (e.g., entertainment), low psychosocial risk factors, and elevated protective factors.

2. The longitudinal trajectory analysis (LGMM) will reveal heterogeneous patterns of depression, with most participants showing a trajectory of stable low depression over time or resilience while smaller subsets of participants will evidence trajectories characterized at different points by elevated (i.e., above the clinical cut‐off) depression, most notably during COVID‐19. Moreover, the latter will be statistically associated with membership in the high risk profile of elevated social media coupled with multiple psychosocial risk factors and few protective factors.

## METHODS

### Participants

Participants for the current study were drawn from a larger sample of adolescents recruited from public schools in a rural, lower‐middle‐class community in the southeastern United States. Beginning in 2016, participants engaged in a yearly, ongoing longitudinal study of peer relations and psychosocial adjustment including five waves of assessment. At Time 4 (November/December 2019), some participants also gave permission for future contact by the researchers and were invited to participate in an additional, virtual wave of assessments at Time 5. The current study focusses on participants at Time 5, which took place between May and September 2020. During this period, schools had been closed to in‐person instruction since March due to COVID‐19 restrictions; in mid‐August, some schools in the area reopened while some provided remote learning only. Participating teenagers were rising 10th and 11th graders from three public high schools in the Southeastern US with ages ranging from 14 to 17 years. During this virtual wave, participants with iPhones were invited to objectively report their smartphone usage via taking and submitting screenshots of the Screen Time app. They also completed several questionnaires, some specific to COVID‐19‐related stress. Out of 242 participants who were enrolled at Time 5, only those participants who correctly submitted their smartphone use data were included in the present study to identify distinct profiles of smartphone use and either psychosocial risk and/or protective factors (*n* = 169). Data of participants included in the present study were collected between May 21th and August 27th 2020. School years in the US districts where the study was conducted end between late May and early June, and start back in mid to late August. This means, that the vast majority of the data presented here were gathered during summer vacation. Any school‐related smartphone use is thus not expected to have had a biasing impact on the results of the here presented analyses. For all Time 5 participants who completed the depression assessment, corresponding data on depressive symptoms from previous Times 1–4 were integrated for the subsequent longitudinal trajectory analyses across all five waves (*n* = 236).

### Procedures

During study waves at Times 1–4, trained research staff visited participants' schools and administered assent forms and measures using computer‐assisted self‐interviews. During the virtual wave at Time 5, participants provided assent online and their parents provided consent online. Participants were compensated between $25 and $50, depending on their rate of participation. Only participants who had iPhones (which have the Screen Time app) were able to contribute screenshots of their daily phone use, which they could upload once a day for 14 consecutive days. Participants used the ExpiWell app (https://app.expiwell.com) to upload their phone use screenshots, and study personnel assisted participants in downloading and navigating the app before data collection began. The 2‐week ExpiWell participation took place on both weekends and weekdays, and start days varied across participants. Participants were reminded to complete screenshot uploads via daily notifications. To clean the screentime data, trained research assistants confirmed whether screenshots were uploaded correctly (i.e., screenshots depicted Screen Time app information containing the correct information from the correct day). In addition, they transcribed the number of daily minutes spent in each category and overall phone use. All transcribed numbers were double‐checked for correctness. All procedures were approved by the university institutional review board.

### Measures

#### Smartphone use

For 2 weeks, participants were asked daily to take screenshots of the previous day's smartphone screen time. Participants accessed this data by selecting their iPhone's Settings app, then the Screen Time function, then “See All Activity” and taking screenshots of their overall screen time and their top three most‐used app categories. Apps are automatically grouped into categories in the Screen Time app based on how developers categorize them in the App Store. Although other categories were available, the Social Media, Entertainment, and Creativity categories were the most often reported by our sample and hence used in the present analyses. Remaining categories contained considerable amounts of zero or missing entries, rendering analyses prone to biases. While contents of the Social Media category include all social media platforms (in this sample, mostly TikTok, Instagram, and Snapchat), the Entertainment category includes for example, video streaming services, such as YouTube. Examples for activities categorized as Creativity are apps used to create digital art or music, such as using the camera, the photos app, photo and video editing apps, layout apps, as well as music apps. When prompted, participants uploaded their screenshots directly within ExpiWell. Trained researchers then double‐coded these screenshots and discussed any discrepancies.

#### Psychosocial risk factors

##### Negative emotions due to COVID‐19

Negative emotions due to COVID‐19 were measured via a portion of the *COVID‐19 Emotional Changes scale*, which the authors developed to explore the impact of COVID‐19 on participants. On a scale from 1 (not at all) to 6 (extremely), participants noted the extent to which they felt 12 negative emotions (e.g., “Anxious”, “Angry”, “Bored”, “Disappointed”) “in the past two weeks, including today, because of the COVID‐19 outbreak, and resulting changes to daily life”. Internal consistency was excellent in the present sample (α = .92).

##### Stress and worry due to COVID‐19

Stress and worry due to COVID‐19 were measured via a portion of the *COVID‐19 Emotional Changes scale*, which the authors developed to explore the impact of COVID‐19 on participants. On a scale from 0 (not at all) to 5 (extremely), participants rated how stressed (3 items) and worried (4 items) they have been in the past 2 weeks due to COVID‐19, the changes it has caused, and its potential health impacts (e.g., “COVID‐19 presents a lot of uncertainty about the future. In the past 2 weeks, including today, how stressful have you found this uncertainty to be?”, “During the past two weeks, how worried have you been about being infected”?). Internal consistency was good in the present sample (α = .87).

##### Perceived stress

Perceived stress was measured via the *Perceived Stress Scale* (Cohen et al., 1983). Ten items on a scale from 0 (never) to 4 (very often) assess the frequency that participants have experienced stress in the last month (e.g., “How often have you been angered because of things that were outside of your control?”, “How often have you found that you could not cope with all the things that you had to do?”); higher scores reflect greater stress. The PSS has demonstrated acceptable psychometric properties according to a review of 19 articles using it (Lee, 2012). Internal consistency was adequate in the present sample (α = .75).

##### Risk‐taking behaviors

A modified version of the *Adolescent Risk Taking Scale* (Alexander et al., 1990) was used to measure how often adolescents have ever engaged in risky behaviors. Eleven items were added to the original version relevant to more modern risk‐taking. Adolescents responded to 17 items using a 4‐point scale from 0 to 4 (never, once or twice, several times and many times) to indicate the frequency with which they have engaged in risk‐taking behaviors, for example, stole or shoplifted, willingly rode in a car with someone who was a dangerous driver, had sex with someone they just met, got drunk or high at a party, tagged or defaced public property, or sent sexy messages or pictures to someone. Internal consistency was good in the present sample (α = .87).

##### Negative interactions with parents and peers

Negative interactions with parents and with peers were measured using 12 items from the *Network of Relationships Inventory: Relationship Qualities Version* (Furman & Buhrmester, 2009). Two sets of six items each, formulated for either a parent or their best friend, were used to measure negative interactions with parents separately from negative interactions with peers. Adolescent were asked how often negative interactions take place in general, without specifying a specific timeframe. Respondents rated items from 1 (little or none) to 5 (the most) (e.g., “How often do you and your best friend disagree and quarrel with each other?”, “How often do you tell your parent everything that you are going through?”, “How often do you and your parent go places and do things together?”). Negative interactions include disagreements, annoyances, fights, and nagging; but also less positive qualities, such as common activities, taking care of each other, or treating the adolescent like they are admired and respected. Higher scores reflect greater conflict. Internal consistencies were adequate (α = .75) and good (α = .87) in the present sample for negative interactions with parents and peers, respectively.

##### Depression

Depressive symptoms were measured via a modification of the *Short Mood and Feelings Questionnaire* (Angold et al., 1995). The scale includes 13 items on a scale from 0 (not true) to 2 (mostly true) which assess the general frequency of depressive symptoms in the past 2 weeks (e.g., “I felt miserable or unhappy”). A total score of 8 or more is considered clinically significant (Messer et al., 1995). In the present study, four items were omitted after Time 1 to speed up data collection. The sum of the remaining nine items indicates more frequent depressive symptoms and, consequently, a total score of 5.5 is considered to be the cut‐off for clinical relevance. Reliability for the 13‐ versus 9‐item versions were very similar (α = .912 and .911, respectively) for the Time 1 sample. The original SMFQ has demonstrated good unidimensionality and reliability (Messer et al., 1995). Internal consistency in the present sample was excellent across waves 1–5 (α = .90–.94).

#### Psychosocial protective factors

##### Support from parents and peers

The general level of support adolescents received from either parents or from peers was measured separately using seven items each from the *Network of Relationships Inventory: Relationship Qualities Version* (Furman & Buhrmester, 2009). As described above for *Negative Interactions with parents and peers*, respondents rate items from 1 (little or none) to 5 (the most) (e.g., “How much does your best friend really care about you?”). Support includes shared enjoyment, instrumental support, emotional intimacy, respect, and care; these interactions with parents were measured separately from interactions with peers. Higher scores reflect greater support. Internal consistencies were good for support from parents (α = .89) and peers (α = .85) in the present sample.

### Statistical analyses

Descriptive statistics for smartphone use variables included computing skewness and kurtosis. In addition, group difference tests across gender were conducted for all Time 5 variables included in the person‐centered analyses. Since several variables' distributions were skewed, robust tests were conducted using bootstrapping (2000 iterations). False discovery rate was used to adjust for multiple comparisons.

Latent profile analysis (LPA) was used to identify groups of adolescents who showed similar patterns of smartphone use and psychosocial risk and/or protective factors, including COVID‐19‐related negative emotions, stress, and worries, perceived stress, risk‐taking behaviors, as well as negative interactions with and support by parents and best friends. LPAs were performed with the *mclust* package (Scrucca et al., 2016) in *R* Version 4.1.3 (R Core Team, 2021). Based on finite Gaussian mixture modeling, LPA models were fit in a series of steps starting with a one‐profile model and subsequently increasing the number of profiles until there is no further improvement in the model. The *mclust* package allows to fit profiles with covariances differing in their distribution structure type, volume, shape, and orientation (Scrucca et al., 2016). Based on simulations by Nylund et al. (2007), we adopted the Bayesian information criterion (BIC) and the Bootstrap Likelihood Ratio Test (BLRT) as the best indicators to determine the optimal number of profiles in the studied sample. For the BIC, in *mclust*, higher values represent better fitting models. The BLRT compares model fit between *k*‐1 and k profile models. A *p* < .05 indicates that the specified model *k* provides better fit to the data than the model with one profile less. We also compared values of the Integrated Completed Likelihood (ICL; Bertoletti et al., 2015) to choose the number of profiles. Finally, entropy was assed, which refers to average accuracy in assigning individuals correctly to the appropriate profiles. Entropy values range from 0 to1, with values from 0.80 being considered high (Clark & Muthén, 2009). All scores have been standardized to allow for better comparability of the different variables ranging on different scales. Missingness in all study variables included in the LPA was low (2.6% total). Missing data were imputed with K–nearest neighbor imputation using the *DMwR2* package in R (Torgo, 2016). Robust tests for profile comparisons were conducted using bootstrapping (1000 iterations) of means, *F*‐tests, and post‐hoc pairwise comparisons (Sidak‐adjusted for multiple comparison).

Latent growth mixture modeling (LGMM), a form of unsupervised machine learning, was performed using *Mplus* Version 7.4 (Muthén & Muthén, 2012) to identify trajectories of depression from Time 1 to Time 5. We explored intercept, slope, and quadratic parameters as either random or fixed effects. Quadratic terms were included to examine whether some trajectory patterns could be better characterized by a parabolic curve than a linear model. In the final models, both slope and intercept variances were allowed to be freely estimated. When including a quadratic term, the model did not converge. Therefore, the quadratic term was excluded in all further analyses. Model solutions from 1 to 4 classes were compared by model fit indices, including Akaike (AIC), Bayesian (BIC), and sample‐size adjusted Bayesian Information Criteria (SSABIC), Entropy, and Lo–Mendell–Rubin likelihood ratio test (LMR‐LT).

Lastly, to empirically describe the distribution of individuals from the depression trajectories across the at‐risk profiles, multinomial logistic regression analyses evaluated the differential odds for being represented in the resultant profiles based on depression trajectory membership.

## RESULTS

### Sample characteristics and descriptive statistics

Demographic characteristics are presented in Table 1. Participants were on average 15.95‐years‐old (SD = .60), predominately female (59.8%) and racially and ethnically diverse. The average median household income for participants' zip codes based on census data at the beginning of the larger study was $44,096.99.

**TABLE 1:** Demographic characteristics.

		*N*	%
Sex	Female	101	59.8
	Male	64	37.9
	NA	4	2.4
Race	American Indian or Alaska Native	3	1.8
	Asian or Asian American	1	0.6
	Black or African American	33	19.5
	White	59	34.9
	Multi‐Racial	17	10.1
	Unclear/Unknown	3	1.8
	Not reported	50	29.6
	NA	3	1.8
Ethnicity	Hispanic or Latinx	51	30.2
	Not Hispanic or Latinx	115	68.0
	NA	3	1.8

Abbreviation: NA, not available.

Table 2 presents the descriptive statistics of the outcome variables included in the present study. Skewness values indicated right‐skewed distributions for the vast majority of the variables. Further, kurtosis values greater than 3 also indicated nonnormal distributions for most variables. Adolescents spent on average about 9 h on their smartphones per day, much of the time using social media (for approx. 3 h). Activities from the entertainment and creativity categories were used less frequently, with about 2 h each per day. Bootstrapped gender difference tests adjusted for multiple testing indicated significantly greater use of entertainment for boys. Examining all time 5 psychosocial variables, girls reported significantly more COVID‐19‐related negative emotions, stress, and worries; greater perceived stress in general; more negative interactions with their parents; greater support by their best friend; as well as more elevated levels of depression.

**TABLE 2:** Descriptive statistics.

	*Skewness*	*Kurtosis*	*Entire sample*				Girls	Boys	*p* ^a^	95% CI of mean difference^b^
			Min	Max	*M*	SD	M(SD)	M (SD)		
Total smartphone use (minutes/day)	0.25	3.47	0	1070.80	543.36	186.64	548.52 (190.28)	526.86 (177.57)	.505	−78.69, 36.05
Social media (minutes/day)	0.92	3.59	6	706.00	198.55	128.54	212.15 (190.28)	181.39 (177.57)	.231	−69.81, 10.92
Entertainment (minutes/day)	2.42	10.49	7	587.46	112.57	103.13	93.59 (73.60)	132.87 (121.46)	.026	8.65, 70.93
Creativity (minutes/day)	1.41	6.39	3	507.00	124.46	84.30	125.10 (85.42)	119.81 (85.28)	.730	−35.00, 25.34
COVID‐19 negative emotions	0.49	2.31	12	64.00	30.94	13.29	34.59 (13.25)	24.71 (10.94)	<.001	−13.98, −5.70
COVID‐19 stress and worry	0.36	2.43	0	30	12.22	7.47	13.70 (7.07)	9.71 (7.28)	.002	−6.35, −1.66
Perceived stress	0.18	3.34	2	36	16.77	6.23	18.10 (6.21)	14.11 (5.32)	<.001	−5.87, −2.02
Risk taking	2.42	11.27	0	49	7.12	7.37	6.46 (5.34)	7.89 (8.96)	.293	−0.66, 3.66
Negative interactions parent	0.83	3.32	24	42	29.37	4.53	30.24 (4.75)	28.05 (3.93)	.006	−3.62, −0.75
Support Parent	−0.16	2.66	29	56	45.08	5.97	45.48 (5.97)	44.66 (6.02)	.475	−2.66, 1.04
Negative interactions best friend	2.07	8.83	24	48	27.65	4.21	27.90 (4.13)	27.33 (4.37)	.475	−1.85, 0.78
Support best friend	−0.46	2.69	28	56	45.81	6.39	47.14 (5.64)	44.30 (6.88)	.010	−4.87, −0.85
Depression	1.05	3.28	0	26	6.82	6.85	7.94 (7.30)	4.35 (4.77)	.002	−5.67, −1.46

^a^ *p* values for *t*‐tests adjusted for multiple comparison using false discovery rate.

^b^ Bootstrapped CIs presented (2000 iterations).

### Latent profile analysis

The LPA included smartphone use variables (total smartphone use, minutes spent with social media, entertainment, and creativity) along with both psychosocial risk and protective factors (negative emotions, and worries related to COVID‐19, perceived stress, risk‐taking behaviors, and quality of relationships with peers and parents). The fit indices of the LPA indicate that the best‐fitting model consisted of three profiles (*BIC* = −5508.28, ICL *= −*5518.46) using an EVE model with ellipsoidal distribution, equal volume, variable shape, and equal orientation. The model with two profiles had higher (i.e., less well‐fitting) BIC and ICL values than the three‐profile solution (*BIC* = −5528.60, *ICL* = −5543.46). The BLRTs were significant for the two‐ and three‐profile solution (*ps* < .001), but not for the four‐profile solution (*p* = .769), suggesting that the three‐profile solution provided the best fit. Additionally, the excellent entropy value of the three‐group solution, 0.974, indicated high accuracy in profile separation, supporting the resulting latent profiles. More specifically, average probability of latent profile membership was high, which implies good discrimination between the three profiles: 0.978 for Profile 1, 0.986 for Profile 2, and 0.957 for Profile 3.

**FIGURE 1:**
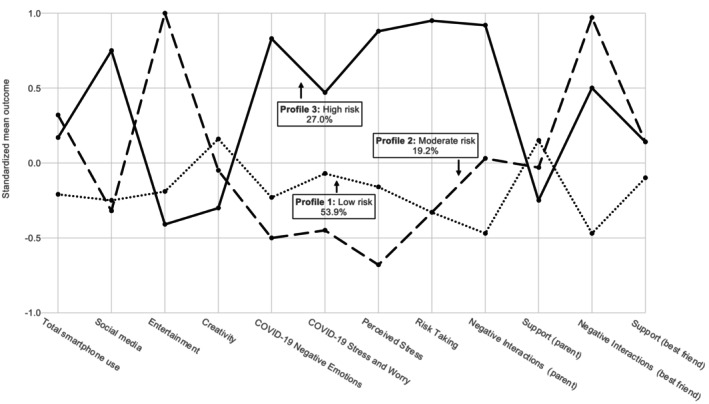
Standardized means for all variables used in the latent profile analyses for the three resultant profiles.

Consistent with our first hypothesis, three‐profile LPA revealed a large low risk group as well as a smaller but critical high risk group. As can be seen by the standardized mean outcomes displayed in Figure 1 and by the differences between variables across profiles, detailed in Table 3, Profile 1 (labeled *low risk*) consisted of 91 adolescents, a majority of the sample (53.8%). Adolescents in Profile 1 were characterized by the lowest levels of total smartphone use compared to Profiles 2 or 3. Similarly, Social media and Creativity use were significantly lower in Profile 1 compared to Profile 3 (no significant differences to Profile 2). This smartphone use pattern was paired with psychosocial risk factors that were predominantly lower than respective scores for Profiles 2 and 3, but with protective factors, that is, social support variables, only trending toward significance or not being significantly different from Profiles 2 and 3. Profile 3 indicated a high risk group consisting of 46 adolescents (27.2%). Adolescents from Profile 3 (labeled *high risk*) were characterized by elevated smartphone use, especially by the highest numbers of minutes spent using social media (i.e., significantly higher than for Profiles 1 and 2), combined with significantly higher scores on all psychosocial risk factors (significantly elevated COVID‐19‐related negative emotions, stress, and worries, greater perceived stress, greater risk‐taking, and more negative interactions with both parents and best friends) and slightly lower, but statistically comparable, scores on protective factors (support by parents and best friends) compared to Profiles 1 and 2 (Table 3). Profile 2 indicated a moderate risk group containing 32 adolescents (18.9%) defined by high smartphone use, mostly due to significantly greater use of entertainment compared to Profiles 1 and 3. Highest entertainment use was paired with relatively lower levels of many risk factors compared to both the high and the low risk Profiles 1 and 3. While most Profile 2 scores on psychosocial risk and protective factors were similar to the low risk Profile 1 (i.e., no statistically significant difference), except for perceived stress and negative interactions with parents and a best friend, Profile 2 risk factor scores were all significantly lower than those in Profile 3. There were no significant differences for the protective factor of parental and peer support between Profile 2 (labeled *moderate risk*) and Profile 3. The moderate risk Profile 2 showed the highest levels of negative interactions with a best friend, compared to both Profiles 1 and 3 (low and high risk).

**TABLE 3:** Profile differences for variables included in the latent profile analysis.

Omnibus MANCOVA	Profile 1 (*n* = 91)			Profile 2 (*n* = 32)			Profile 3 (*n* = 46)			Post‐hoc comparisons^a^		
*F*(24,312) = 24.74, *p* < .001	M	Bootstrap SE	Bootstrap 95% CI	M	Bootstrap SE	Bootstrap 95% CI	M	Bootstrap SE	Bootstrap 95% CI	Profile 1 versus 2 *p*‐value	Profile 1 versus 3 *p*‐value	Profile 2 versus 3 *p*‐value
Total smartphone use	505.90	17.79	471.75 542.50	599.83	37.84	523.20 673.00	578.18	25.89	524.77 628.60	.040	.098	.940
Social media	166.44	11.12	145.68 189.01	148.04	17.37	117.80 183.35	295.46	20.45	257.88 336.03	.815	<.001	<.001
Entertainment	92.45	5.92	81.86 104.77	221.42	28.35	168.31 279.75	71.743	5.97	60.56 84.07	<.001	.437	<.001
Creativity	139.21	9.79	121.39 159.59	119.97	8.31	104.10 136.74	100.97	7.04	88.16 115.15	.129	.006	.079
COVID‐19 negative emotions	27.81	1.18	25.62 30.20	24.03	1.64	20.90 27.31	42.15	1.93	38.33 45.84	.071	.001	.001
COVID‐19 stress and worry	11.63	.81	10.13 13.272	8.91	.91	7.25 10.78	15.70	1.03	13.64 17.80	.179	.005	<.001
Perceived stress	15.82	.55	14.82 16.94	12.63	.88	10.90 14.26	21.54	.88	19.79 23.21	.012	<.001	.001
Risk taking	4.63	.38	3.91 5.40	4.38	.87	2.87 6.32	13.92	1.38	11.38 16.92	.996	<.001	<.001
Negative interactions parent	27.22	.32	26.62 27.85	29.56	.67	28.30 30.97	33.50	.69	32.15 34.85	.007	<.001	<.001
Support Parent	44.88	.59	44.82 47.10	44.97	1.04	43.00 47.12	43.57	.94	41.74 45.41	.839	.094	.664
Negative interactions best friend	25.69	.20	25.28 26.08	31.89	1.10	29.77 33.95	28.57	.49	27.58 29.59	<.001	<.001	<.001
Support best friend	45.08	.69	43.77 46.38	46.69	1.20	44.24 48.91	46.65	.79	44.98 48.12	.527	.436	1.000

Abbreviation: SE, Standard error.

^a^ Post‐hoc comparisons based on Sidak‐adjusted bootstrap pairwise comparisons (1000 iterations).

### Identification of depression trajectories

Table 4 displays the fit statistics for each depression trajectory. We examined one‐ to four‐class‐solutions. Information criteria (AIC and SSABIC) decreased consistently, indicating improved model fit with increasing class number. Entropy was high for each selected solution (>.80) and varied only slightly between the different models. LMR‐LRTs, which compared each class solutions with the previous (n – 1) class solution, were significant for three classes but not four. BLRT was significant for the four‐class solution, but the percentage of participants in the smallest class was very low (6%). Thus, the three‐class solution was selected as optimal.

**TABLE 4:** Fit Indices and Entropies for Latent Growth Mixture Models.

Nb of classes	Maximum LL	AIC	SA‐BIC	LMR‐LRT *p*‐value	BLRT *p*‐value	Entropy	% smallest
1	−3510.46	7042.92	7046.20	‐	‐	‐	‐
2	−3073.48	6178.96	6183.74	<.001	<.001	.88	23%
3	**−2987.05**	**6016.09**	**6022.36**	**<.001**	**<.001**	.**86**	**9%**
4	−2962.54	5977.08	5984.84	.44	<.001	.87	6%

*Note*: A significant test indicates that a solution with a given number of classes provides a better fit to the data than a solution with one fewer class. % smallest class = percentage of participants in the smallest group. Best‐fitting model indicated in bold.

Abbreviations: AIC, Akaike information criterion; BLRT, parametric bootstrapped likelihood ratio test; LMR‐LRT, Lo–Mendell–Rubin likelihood ratio test; Maximum LL, maximized log likelihood value; SA‐BIC, sample‐size adjusted Bayesian information criterion.

**FIGURE 2:**
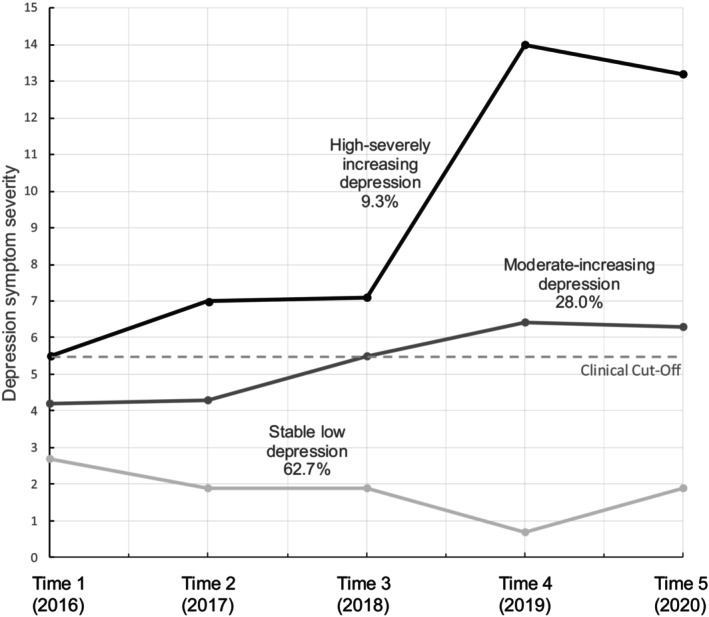
Group means for depression trajectories.

As can be seen in Figure 2, a majority of adolescents (*n* = 148, 62.7%) were characterized by a trajectory of *stable low depression* or resilience across all five waves. The means for this group fell well below the clinical cut‐off at each time point. The next most prevalent trajectory (*n* = 66, 28.0%), *moderate‐increasing depression*, was characterized by sub‐threshold depression at Times 1 and 2 that gradually increased to meet the clinical cut‐off at Time 3 and then exceed the clinical cut‐off at Times 4 and 5. Finally, a third trajectory (*n* = 22, 9.3%), *high‐severely increasing depression* was characterized by depression at the clinical cut‐off at Time 1, increases to above threshold depression at Times 2 and 4, then spiking to highly elevated depression at Time 4 and dropping slightly to remain highly elevated at Time 5.

### Locating individuals in risk profiles according to their depression trajectories

**FIGURE 3:**
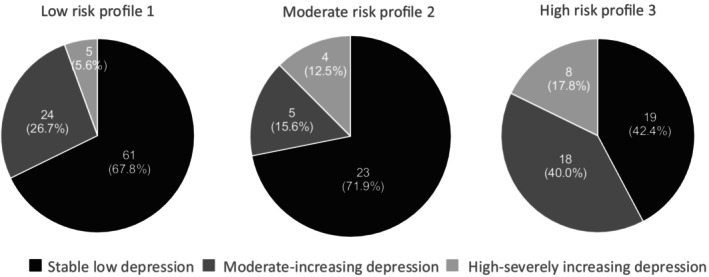
Distributions of teenagers in the three depression trajectories across the three latent risk profiles.

Consistent with our second hypothesis, Figure 3 indicates, adolescents with elevated depression before and during the COVID‐19 were more likely to be in the high risk profile. More specifically, as Figure 3 illustrates, both the moderate‐increasing and high‐severely increasing trajectories were more prevalent in the high risk group (Profile 3), than in the moderate (Profile 2) or low risk (Profile 1) groups. Table 5 shows that when compared to the low risk profile, the odds for teenagers from both the high‐severely increasing and the moderate‐increasing depression trajectories to be represented in the high risk profile were 5.14 and 2.41 times greater (respectively) than for teenagers from the stable low depression trajectory. However, adolescents from neither the moderate‐increasing nor the high‐severely increasing depression trajectories (compared to those from the stable low depression trajectory) had significantly greater odds of being in the moderate risk profile (compared to the low risk profile, see Table 5). Further, although not displayed in Table 5, no significant differences were found between the odds of adolescents from the high‐severely increasing versus adolescents from the moderate‐increasing depression trajectories regarding their membership in the high risk profile or the moderate risk profile (versus the low risk profile; Exp(B) = 2.13, 95% Wald CI 0.60, 7.62, *p* = .244 and Exp(B) = 3.84, 95% Wald CI 0.75, 19.60, *p* = .106, respectively).

**TABLE 5:** Multinomial logistic regression analyses for depression trajectories predicting latent profiles.

Trajectory	Moderate‐risk profile 2^a^			High‐risk profile 3^a^		
	Exp(B)	95%CI^b^	*p*‐value	Exp(B)	95%CI^b^	*p*‐value
Moderate‐increasing depression^c^	0.55	0.19, 1.62	.280	2.41	1.08, 5.35	.031
High‐severely increasing depression^c^	2.12	0.52, 8.60	.292	5.14	1.50, 17.58	.009

^a^ The low‐risk profile 1 serves as reference class.

^b^ 95% Wald CI.

^c^ The stable low depression trajectory serves as reference class.

## DISCUSSION

In this study, we examined profiles of objectively assessed smartphone use coupled with psychosocial risk and/or protective factors in a sample of US adolescents captured during the COVID‐19 pandemic. LPA led to three empirically derived profiles that could be categorized from low to high risk according to varying patterns of smartphone activities coupled with different sets of psychosocial variables. Consistent with our first hypothesis, although the majority of adolescents (53.8%) were captured by a low risk profile, a small but critical subset (18.9%) fell into a high risk profile characterized by elevated social media use, high levels of psychosocial risk factors, and low levels of protective variables. To test our second hypothesis, we created trajectories of depression symptoms from 5 yearly waves of data beginning prior to and culminating in the first year of the pandemic. This analysis identified three distinct depression trajectories: stable low depression, moderate‐increasing depression, and high‐severely increasing depression. Consistent with hypothesis 2, both the moderate‐increasing and high‐severely increasing trajectories, which were characterized by elevated depression prior to and during COVID‐19, were significantly associated with membership in the high risk profile.

A key implication to emerge from the LPA was that smartphone use per se does not necessarily impact adolescents' mental health, even when they spend multiple hours a day on their phones. Instead, that impact was more clearly linked to the kind of smartphone activity adolescents engage in. For example, the high versus the moderate risk profiles were not distinguished by the total number of minutes adolescents spent on their smartphone, but rather by different use patterns for social media and entertainment. The adolescents in the high risk profile used social media the most but also reported a variety of elevated psychosocial risk factors, that is, elevated COVID‐19‐related negative emotions, stress, and worries, above‐average perceived stress, greater risk‐taking, and more negative interactions with both parents and best friends, and low protective factors, that is, lower support by parents and best friends. By contrast, adolescents in the moderate risk profile used entertainment the most (and had very low usage of social media), but did not report elevated levels of risk factors, with the exception of increased negative interactions with their best friends. The relevance of these findings to adolescent behavior and mental health is pointed out by the fact that while most adolescents were categorized in the low risk profile, a considerable portion of almost 30% were nonetheless represented in the high risk profile.

A particularly important implication of the high risk profile is that it combined elevated social media use with reports of negative emotions, worries regarding the COVID‐19 pandemic, and increased levels of stress. While this finding is compatible with existing evidence indicating a positive association between adolescent social media use and decreased well‐being (e.g., Schønning et al., 2020), it also warrants further discussion in the specific context of the COVID‐19 pandemic. On the one hand, it could be argued that social media was used a lot during the pandemic since it could function as a distraction from stressful encounters (reviewed in Wolfers & Utz, 2022), which could have been adaptive for adolescents from the present study who were prone to worry or experienced high levels of stress. On the other hand, elevated social media use may serve as a platform for expressing and sharing their thoughts and feelings (Boyd & Ellison, 2007) which, in the context of COVID‐19, could highlight fears or concerns with social restrictions. As a consequence, spending a lot of time on social media might lead to ruminative processes which could, in turn, enhance negative feelings and worries around the pandemic. Social media can also be used to spread misinformation (Hopp et al., 2020), which again, could foster negative feelings and fears around COVID‐19. On balance, our results clearly align with findings of a recent study in adults showing that problematic social media use was associated with higher COVID‐19‐related stress and specifically with the use of social media for coping (Moretta et al., 2023). This could also explain the co‐occurrence of elevated levels of COVID‐19‐related worries and stress with high rates of social media use in the present study.

Surprisingly, adolescents in the moderate risk profile, characterized by elevated use of entertainment and mostly low levels of psychosocial risk factors, indicated greater negative interactions with their best friends, as compared to adolescents from the other two profiles. Research in adolescents and young adults has shown an association between gaming and social withdrawal (e.g., Fong et al., 2023). It may be that, similarly, adolescents who spend much of their time streaming videos on YouTube or Netflix (categorized as Entertainment in the Screen Time function) also withdraw socially. As such, decreased interaction with peers may be linked to lower quality of relationship and/or greater conflict.

Framing our study into the context of theoretical models, the DSMM (Valkenburg & Peter, 2013), aligns well with our LPA findings. While we cannot speak to effects of social media use, as suggested by the DSMM, owing to the cross‐sectional nature of our results, our findings emphasize the integration of both media (i.e., smartphone use) and nonmedia variables (i.e., individual differences variables, social context) and provide information about their relationships as proposed by the DSMM. As such, our LPA included a comprehensive set of both smartphone use variables as well as individual psychosocial variables and our results highlight underlying patterns linking these two sets of variables in different ways. The results presented here are also well in line with the I‐PACE model (Brand et al., 2019) and the *transformational framework* (Nesi et al., 2018) since they emphasize the role of predisposing and mediating factors in elevated social media use, including affective (assessed in the present study via negative emotions and stress) and cognitive variables (i.e., worries), a lack of self‐regulatory behavior (i.e., risk raking) as well as peer relationships (i.e., interactions and support).

In general, our findings about the three trajectories of depressive symptoms are comparable to findings of previous studies longitudinally modeling depression symptom trajectories in adolescent as they overlap very well with common subgroups that represent heterogeneity in the development of depression (reviewed in Shore et al., 2018). Existing literature has also shown that the prevalence of depression increases markedly between the ages of 12 and 15 years (Kessler et al., 2001). A recent trajectory study revealed a subgroup of adolescents displaying a steep increase of depressive symptoms around the age of 14 years (Lussier et al., 2021). This aligns well with the high‐severely increasing trajectory of the present study, showing a dramatic spike in depression symptoms at Time 4 when participants were between 13‐ and 16‐years‐old.

One exception in the current study, however, is that we did not observe the increase in depression that has been repeatedly reported amongst adolescents during the COVID‐19 pandemic. One potential explanation could be, that the effects of COVID‐19 on adolescent mental health were not yet “visible” at Time 5, since data were collected relatively early in the pandemic (starting in May 2020). Stay‐at‐home orders in North Carolina were issued in March 2020. During the very first months of school closures, adolescents from the present study might not yet have experienced a strong negative impact on their well‐being, assuming that they were initially adapting well to many activities taking place remotely and, in turn, some might even have been relieved to not have to go to school. A recent study showed that school closures and exam cancellations did not impact perceived changes in depression as negatively as COVID‐19 per se (Stewart et al., 2022). Finally, Workman et al. (2023) found that, with adolescents' and young adults' number of depression diagnoses generally increasing between March 2018 and March 2022, they only peaked later during the pandemic, that is, in March 2021. Finally, a study including a large sample of adolescents between 13 and 16 years in Norway showed that depressive symptoms increased slightly between 2019 and 2020, but that change was no longer significant when controlling for age. Hence, these authors concluded that the increase in depression were not due to effects of the pandemic, but rather to age (Hafstad et al., 2021). It is plausible that a similar age effect, rather than COVID‐19 per se, might also explain the increase of depression observed in the present study.

While it may seem intuitive that increased depression over time indicated a subgroup of adolescents characterized by elevated levels of worries, stress, and lowered quality of relationship with caregivers and peers, this group's use of their smartphones for social media is particularly interesting. Vulnerable groups of teenagers, such as adolescents suffering from depression, may be more prone to engaging in social media for seeking social support to potentially ease their strain and worries (Liu et al., 2018; Selkie et al., 2020). This is also in line with theoretical assumptions, such as the DSMM (Valkenburg & Peter, 2013), suggesting that depression represents a predisposing or susceptibility variable impacting social media use. On the contrary, adolescents reporting high levels of depressive symptoms might be especially prone to fear of missing out (FOMO) and comparison with peers. Thereby, social media might hold their attention particularly well as it aligns with their worries and ruminations (reviewed in Fioravanti et al., 2021). For individuals suffering from mental health difficulties, social media can also serve as a medium to seek for more information about mental health in order to facilitate coping (Akhther & Sopory, 2022). Finally, depressed adolescents could also be engaging less in in‐person social activities and thus benefit from using social media to connect with others online.

In summary, our findings showed that during COVID‐19, for a considerable number of adolescents, elevated social media use was linked to elevated risk factors, including indicators of mental health difficulties, and lowered protective factors. We discussed the explanation of using social media for coping for example, with worries, stress or mental health difficulties, being particularly relevant during a stressful period as COVID‐19. Our findings also highlight the particular strain COVID‐19 has put on girls since, compared to boys, they reported greater negative emotions, stress and worries due to COVID‐19, higher levels of depression and more negative interactions with their parents at Time 5. All this is in line with current discussions about youth mental health. While experts are alarmed by a surge in already elevated levels of poor youth mental health due to COVID‐19 and related restrictions (The Lancet Psychiatry, 2024), COVID‐19 has fostered the integration of online mental health tools into daily life and bolstered a digital transformation that has changed where adolescents seek information and access support (Ludwig‐Walz et al., 2024). Our findings therefore underscore the role of social media in youth mental health not only by pointing out potential detrimental associations, but also by emphasizing the opportunity to use social media to promote mental health and well‐being amongst adolescents. Mental care providers should thus reinforce their presence on social media, thriving to provide adolescents easy, fast and flexible access to online mental health services of certified quality and to integrate those into stepped‐care approaches. This is of particular importance since crises like the COVID‐19 pandemic or others like the climate crisis or war present ongoing challenges for today's adolescents.

### Limitations and future research

Unique strengths of this study include the objective assessment of multiple types of smartphone use over a prolonged period in a relatively large, racially and ethnically diverse sample of adolescents during COVID‐19, as well as the use of a person‐centered approach revealing insights into subgroups of adolescents derived from varying combinations of smartphone use and psychosocial risk versus protective factors. However, several limitations need to be taken into account. First, smartphone data were captured during the first wave of the COVID‐19 pandemic. While this provides us with important information about a uniquely stressful time, previous research showed that adolescents' screen time use, in particular social media use, increased during that period (e.g., Viner et al., 2022). During summer 2020, restrictions on social gatherings were in place, which could explain the greater use of social media to stay connected to peers and family members. Social media use has also been shown to be positively related to loneliness (reviewed in O'Day & Heimberg, 2021). For these reasons, it is possible that our results may have overestimated the time adolescents spent on their smartphones and thus require replication.

Second, from a methodological perspective, although our data present a more nuanced picture of what adolescents do on their smartphones by objectively assessing the time, they spent in specific apps rather than solely focusing on total time using their phone, time does not necessarily reflect specific behaviors or experiences. Future research will need to fill this gap in order to gain more insight into whether specific behaviors and motivations, such as for example using smartphones for social comparison and feedback‐seeking (Nesi & Prinstein, 2015) or to alter/avoid negative feelings (Gingras et al., 2023), have beneficial or detrimental impacts on social and emotional functioning in adolescents. Importantly, today's adolescents face unique risks online, as smartphones, and in particular social media, opens myriad avenues to engage in risky activities, potentially exposing them to possible online‐initiated exploitation and victimization, such as grooming, sexual solicitations, and cyberbullying (Craig et al., 2020; Forni et al., 2020; Noll et al., 2021). Such at‐risk online behaviors include sharing personal information, conversing with strangers (Mitchell et al., 2007), meeting with strangers offline whom adolescents had first met online (Noll et al., 2021), provocative online self‐representation (Noll et al., 2009), and being exposed to sexually explicit contents (Haag et al., 2022; Maas et al., 2019).

Third, our study did not capture parental control and monitoring of participants' smartphone use, which may have been a confounding factor. Lastly, the sample only included adolescents who use iPhones and a considerable portion of unreported race (29,6%). This was because *Hispanic/Latino* ethnicity was not listed separately from racial categories. Future studies can increase generalizability by tracking the use of a wider range of smartphone devices as well as gather more fine‐grained information about ethnicity and race.

Lastly, despite our findings, it is important to acknowledge the potential conceptual overlap between depressive symptoms and negative emotions, negative social interactions, and perceived stress. While our analysis was based on longitudinal trajectories of depression prospectively predicting risk profiles, the cross‐sectional nature of the risk profile data at Time 5 limits our ability to fully disentangle their effects. Future research should aim to collect and analyze longitudinal data on social media and psychosocial factors to examine their concurrent and prospective relationships with depression. This approach would allow for a more nuanced understanding of how negative affective indicators and social media use patterns contribute to depressive symptoms over time.

In terms of clinical implications, findings from the LPA can help identify risk profiles of adolescents. Our findings highlighted a subgroup of at‐risk adolescents who spent large proportions of their days on social media, who experienced high levels of worries and stress, reported risky behaviors as well as a lowered quality of relationship with caregivers and peers. Overall, the findings underscore the clinical importance of closely monitoring and possibly intervening on heavy social media use. In particular, adolescents with a history of depression should be considered for possible prevention and intervention programs focusing on social media usage.

## CONCLUSIONS

The present study showed that although high risk and moderate risk profiles did not differ significantly in total time the adolescents spent on their smartphones, these groups did show different patterns of smartphone activities. Elevated social media use was associated with increased psychosocial risk factors and lowered protective factors, whereas elevated entertainment use was mostly associated with lower levels of risk factors. This difference highlights the potential detrimental associations of elevated social media usage and well‐being amongst adolescents. Further, trajectories of increasing depression over time were associated with membership in the high risk profile. Together, these findings describe a group of adolescents who use social media excessively, experience high levels of stress and worries, engage in risky behaviors, report lowered quality of relationship with caregivers and peers and also have been suffering from depression over the past 5 years. Clinical intervention and prevention strategies in adolescents should include and specifically target social media usage, in particular when working with adolescents with a history of depressive symptoms. Importantly, since results pertain to a unique period, that is, COVID‐19, where both digital transformation and mental health difficulties surged, we discuss the potential for using social media for youth online mental health services.

## FUNDING INFORMATION

This work was supported in part by the Winston Family Initiative for the Study of Technology and Adolescent Development, co‐directed by E.H.T. and M.J.P. Any opinions, findings, conclusions, or recommendations expressed in this material are solely the responsibility of the authors and do not necessarily represent the views of this funding source.

## CONFLICT OF INTEREST STATEMENT

The authors declare no conflicts of interest.

## Data Availability

The data that support the findings of this study are available from the corresponding author upon reasonable request.
